# Distribution of Population Sizes in Metapopulations of Threatened Organisms—Implications for Conservation of Orchids

**DOI:** 10.3390/plants14030369

**Published:** 2025-01-25

**Authors:** Zuzana Štípková, Pavel Kindlmann

**Affiliations:** 1 Global Change Research Institute CAS, Bělidla 986/4a, 60300 Brno, Czech Republic; pavel.kindlmann@centrum.cz; 2 Institute for Environmental Studies, Faculty of Science, Charles University, Benátská 2, 12900 Prague, Czech Republic

**Keywords:** conservation, metapopulation, orchids, passive disperser, population size

## Abstract

Species are disappearing worldwide, and it is likely that the rate of their disappearance will increase. The most important factors responsible for this are assumed to be changes in climate and land use. To determine the probability of extinction of a given species, it must be viewed as a metapopulation composed of many populations. In plants, seeds are spread by wind or water (passive dispersers), unlike active dispersers, which can actively look for a suitable site of their species. Thus, while active dispersers can locate a suitable site, passive dispersers often fail to arrive at a suitable site. The following question arises: is it better for the survival of a metapopulation of passive dispersers to concentrate on conserving a few large populations, each of which will produce many propagules, or on many small populations, each of which will produce only few propagules? Here, we address the question of which of these strategies will maximize the likelihood of the survival of such a metapopulation, using orchids as a model. We concluded that small populations should be preferentially preserved. Small populations are more numerous and more likely to occur more widely in the region studied and therefore a larger proportion of the seeds they produce is more likely to land in suitable habitats than that produced by the fewer large populations. For conservation, there is a possibility to extend the results to other taxa. However, this must be carried out with caution and must consider the taxon in question.

## 1. Introduction

Species are disappearing worldwide, and it is likely that the rate of their disappearance will increase in the future [1]. According to the 2019 International Platform for Biodiversity and Ecosystem Services (IPBES), the sixth mass extinction of species is currently underway, reaching up to a hundred times the rate compared to the situation over the last ten million years [2].

The most important factors responsible for this are commonly assumed to be changes in climate and land use [3], with land use often ranked as the most important [3,4]. Land use change, mainly associated with the alteration or even destruction of undisturbed or partially disturbed habitats, is viewed as the main driving force of the decline in biodiversity [5]. This is also the case for orchids [6,7]. It is widely assumed that broad-scale global land-cover transformation will shortly increase species extinction rates [8,9,10,11]. Climate change, however, is also often cited as one of the major driving forces [12,13,14,15,16,17,18,19].

When determining the probability of the extinction of a given species, it must be viewed as a metapopulation composed of many individuals in more or less isolated populations [19,20]. Much attention has been paid to the dynamics of metapopulations, especially when metapopulation dynamics are affected by the migration of individuals between extant populations [19,20].

However, this is not always the case. In many taxa, especially plants, the propagules (seeds) are passively spread by wind or other means (passive dispersers). Orchids, which are used as a model group in this paper, are a good example of a group whose seeds are dispersed passively. Orchid capsules contain characteristic dust-like seeds that can theoretically travel many miles away from the mother plants. However, the distribution curve of the orchid seeds’ dispersal can be described as a heavy-tail one, since the majority of seeds fall in the close vicinity of mother plants, while a few can indeed be dispersed further away [21,22]. The propagules (seeds) are therefore unable to actively choose where they land, unlike, e.g., butterflies, which can actively look for a suitable site that hosts, hosted, or can potentially host a population of their species (active dispersers). Thus, while active dispersers can often find a suitable location, passive dispersers more often do not. To avoid confusion, note that in this paper we use the word “disperser” for an organism that moves, spreads or is transported away from the place of its origin, not for an agent that disperses other organisms (e.g., birds dispersing plant seeds).

The expectation, therefore, other things being equal, is that the proportion of suitable sites located by active dispersers is larger than that located by passive dispersers. In passive dispersers, for which orchids are an example, it is likely that their metapopulation dynamics are of a different and unexplored type, in which, in the course of time, some localities become unsuitable, and others become occupied, in the vast majority of cases where no orchids have ever grown before. To our knowledge, this type of dynamic has been previously studied only once, by Švecová et al. [23], who analyzed data for four orchid species in four regions of the Czech Republic. Their results are very difficult to interpret, as the dependencies of the frequencies of populations on the number of individuals in the population are often contradictory and there is no clear dependence in most cases. This is most likely a consequence of the relatively small number of populations they used rather than the methodology as it was not possible to accurately predict the number of individuals in populations based on the number of flowering individuals.

Here, this analysis is repeated using much larger numbers of populations (all populations of the five most abundant species in the Czech Republic), which yields much better and more definite results.

### Frequency Distribution of Population Sizes in a Metapopulation of a Species with Passive Dispersal

By definition, a *population* is a group of organisms of the same species which inhabit the same geographical area and are capable of interbreeding. *A metapopulation* is a group of such populations which interact at some level. The frequency distribution of population sizes (see https://www.scribbr.com/statistics/frequency-distributions/ for an exact definition of this expression, accessed on 8 December 2024) in a metapopulation can markedly affect its survival. The expectation is that there will be very few populations with large numbers of individuals in a metapopulation of passive dispersers for two reasons:

First, as described above, any population with passive dispersers starts with one individual, which has arrived at the site, unlike populations established by active dispersers, which can exist indefinitely, as newcomers will arrive from other populations. So, for passive dispersers, it takes time to build up a population of many individuals and many disasters can occur during the period of logistic population growth, which can result in the extinction of the population. As the likelihood that a large population will result from one individual is extremely low because of all the negative issues during this process, the number of large populations is likely to be low for passive dispersers.

Second, a large population needs a large area of suitable habitat that will exist for a long time, which is likely to be rare.

On the other hand, with the millions of seeds produced by passive dispersers, one can expect many “mini-populations” consisting of just one individual or of a few of its descendants. Because of the small size of mini-populations, they are unlikely to survive for long; most of them will become extinct and will not become a large population after a long period of uninterrupted logistic population growth. This all leads to an intuitive expectation that the dependence of the number of populations on the number of individuals they consist of will be a declining function and the most likely candidate for this dependence is a negative exponential. This intuitive expectation is tested here.

The crucial question for the survival of a metapopulation of a threatened species is as follows: is it better to concentrate on conserving a few large populations, each of which will produce many propagules, or on conserving many small populations, each of which will produce only few propagules? Which of these strategies will maximize the likelihood of the survival of such a metapopulation?

## 2. Results

The number of populations included the following: 110 for *Cephalanthera damasonium*, 468 for *Dactylorhiza fuchsii*, 1051 for *Dactylorhiza majalis*, 207 for *Neottia ovata* and 529 for *Platanthera bifolia*. Regarding the population sizes (number of individuals in a population), in a very rough approach, a population with less than 30 individuals can be considered as small, a population with more than about 300 individuals may be called large, and anything between 30 and 300 individuals can characterize an intermediate population. However, population size strongly depends on the species, population age, and many other factors. Because we are studying temporal trends in the population sizes, rather than making exact numerical predictions, the exact population sizes are irrelevant in our study.

**Figure 1:**
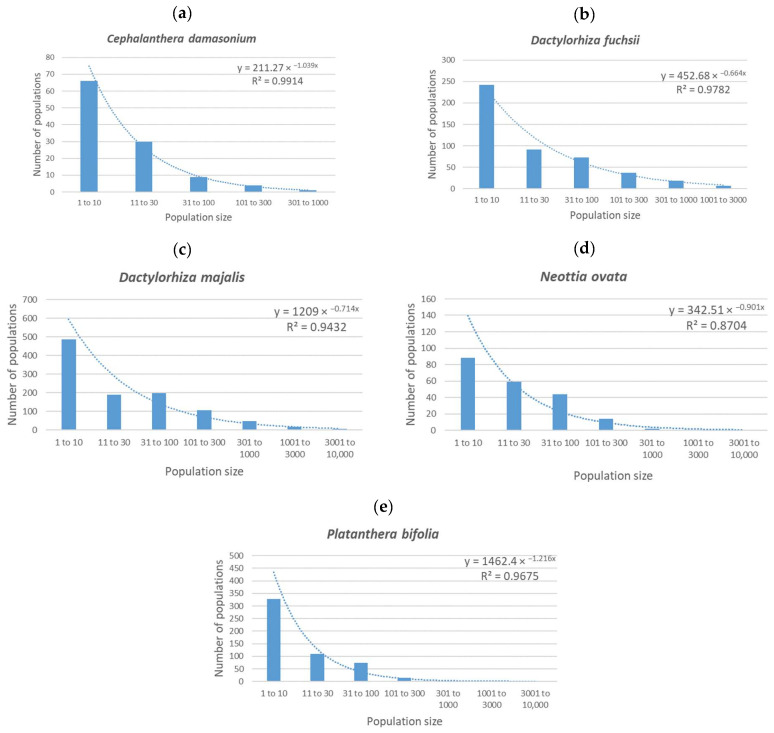
Relationships between population size and number of populations of this size for the five most abundant species of orchid in the Czech Republic: (**a**) *Cephalanthera damasonium*, (**b**) *Dactylorhiza fuchsii*, (**c**) *Dactylorhiza majalis*, (**d**) *Neottia ovata* and (**e**) *Platanthera bifolia*. Clearly, the relationships are all negatively exponential.

In Figure 1, the number of populations of different sizes is displayed for each of the species studied. It is clear that in the graphs (Figure 1a–e), the highest number of populations is for the population size category of 1–10 flowering individuals in all the species studied. Then, the number of populations recorded in the following categories decreases. The only exception is in *D. majalis*, where there are slightly fewer populations with 11–30 flowering individuals than populations with 31–100 individuals (190 and 199 populations, respectively).

When the population size on the horizontal axis is expressed as a decadic log of the mean of each category, then the data show a clear trend of a negative exponential with explained variability between 87% (*N. ovata*) and 99% (*C. damasonium*). To be sure that this is not an error due to the non-random choice of species, the same procedure was followed for 15 additional species that were randomly selected from the dataset. The data for these species show the same trend as was recorded for the five species studied, so it is likely that the same trend will be recorded for all the orchids in the Czech Republic.

These results support the intuitive expectation that the dependences of the number of populations on the number of individuals take the form of a negative exponential and that the different results reported by Švecová et al. [23] were most likely due to the low numbers of populations considered.

### Implication of These Results for Improving Species Conservation

It now remains to determine whether it is better for the survival of a metapopulation to concentrate on conserving a few large populations or many small populations. That is, which of these will give rise to the largest number of propagules (seeds) that when passively dispersed in the landscape will survive and ensure the survival of the species? In other words, which of these strategies will maximize the likelihood of the survival of a metapopulation?

**Figure 2:**
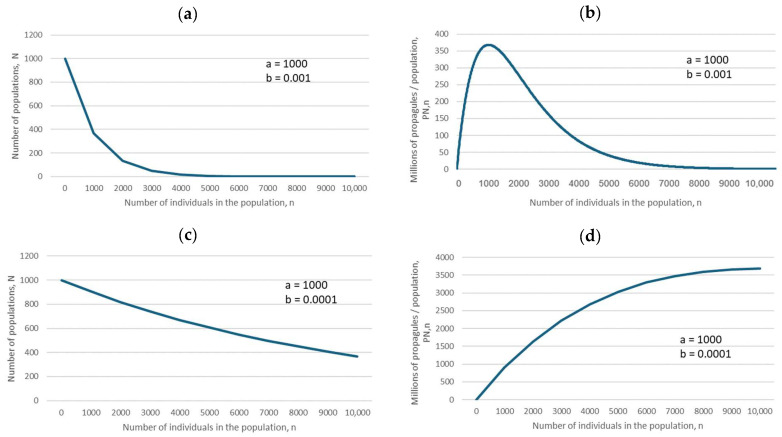
Relationships between the number of populations, *N*, and the number of fertile individuals in a population, *n* (**a**,**c**), and between the number of propagules produced, *P*_N,n_, and the number of individuals in a population, *n* (**b**,**d**), based on Equations (1) and (3) (for descriptions of all equations, see Section 4). The parameter values are in the insets.

The predictions of Equations (1) and (3) (for descriptions of all equations, see Section 4), specific cases of which are illustrated in Figure 2, biologically mean that if the negative exponential is flat, as in Figure 2c, then the maximum number of propagules is produced by large populations, which should therefore be preferentially conserved. However, if the negative exponential declines steeply, as in Figure 2a, then the maximum number of propagules is produced by small populations (because of their large number, compared with that of large populations), and therefore small populations should be preferentially conserved.

**Figure 3:**
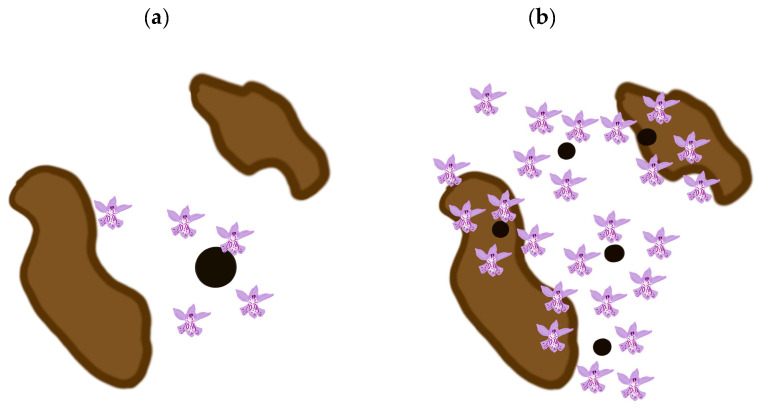
Number of hits (flowers) within and outside suitable habitats (highlighted in brown) for small and large source populations (small and large circles). Large populations are less numerous in the region (see Equation (1) described in Materials and Methods and Figure 1a–e and Figure 2a,c) and because of the negative exponential distribution of distances between the source population and the point where the propagule lands, the number that land in suitable habitats is small (see (**a**)). Small populations are more numerous in the region (see Equation (1) described in Section 4 and Figure 1a–e and Figure 2a,c) and because of the negative exponential distribution of distances between the source population and where the propagules land, the number that land in suitable habitats is larger than that for large populations, because the source populations occur widely in the region studied (see (**b**) and compare the results obtained here with those depicted in (**a**)).

Based on the model presented above, our results suggest that at least in the Czech Republic, it is better to focus our conservation efforts on small populations. However, this management suggestion is not general. The models of seed dispersal [21,22,24,25,26,27,28], etc., predict a negative exponential decline in the number of propagules with an increase in the distance from the source. As depicted in Figure 3, small populations are more numerous and more likely to occur more widely in the region studied and therefore a larger proportion of the seeds they produce is more likely to land in suitable habitats than that produced by the fewer large populations. This of course depends on the spatial distribution of both small and large populations and suitable habitats, which is depicted in Figure 3. Thus, it is difficult to provide general rules for the management of small vs. large populations, as the spatial distribution of both small and large populations and suitable habitats must be considered in each case. That is, the optimum conservation strategy is dependent on local conditions and the species.

## 3. Discussion

There are not a lot of studies on orchid metapopulations. This is probably because it is difficult to accurately map the dispersal of seeds, the colonization of new areas, or to record a population as extinct [23]. In addition, when studying plant metapopulations, other factors should also be considered, such as plant dormancy and the occurrence of sterile individuals [29], the existence of a seed bank, adaptation to changing conditions and limits of seed dispersal [30]. Due to the complicated symbiotic relationships of orchids with other organisms, monitoring and searching for new orchid locations should consider whether their pollinators and mycotrophic fungi are present there [31]. A very good analysis of how the peculiarity of the orchid life cycle (dormancy, seed dispersion, etc.) might have a toll/affect the results was published in [32]. Therefore, orchids can be used as indicators of the state of vegetation [33].

Previously, Švecová et al. [23] conducted a similar study at a smaller scale using the NDOP database. Since there was insufficient data in the NDOP database for orchids in the areas studied, they only had sufficient data for *D. majalis* in South Bohemia and the Bohemian-Moravian Highlands and for *D. fuchsii* in Jeseníky. There was insufficient data for *P. bifolia* and *N. ovata*, as a maximum of nine individuals were recorded and consequently, they only had data for ten localities for these species.

The typical size of an orchid population within a metapopulation is essential for species conservation, as it makes it easier to reach decisions on the need for conservation or appropriate management. The abundance of plants tends to be monitored at individual locations. Records of the number of individuals in a metapopulation should be monitored more than once, because the chance of recording a species during a visit to a site is around 80%; therefore, long-term monitoring is needed [34]. For the reasons stated above, the localities where *D. majalis* occurs were visited repeatedly (in 2021, 2022 and 2023) by Švecová et al. [23] and this should be continued in the future, since long-term field studies are needed, as they provide important information on plant populations [7].

Another question remains: what is the area of sites where orchid populations occur? Švecová et al. [23] report that in the Bohemian-Moravian Highlands, the sites are mainly small, with an area mainly of up to 1 ha. Wotavová et al. [35] report that only 18% of all the areas of sites of *D. majalis* in southern Bohemia are larger than 5000 m^2^. In addition, almost half of all populations occur in waterlogged meadows, which are habitats that are among the most endangered in the Czech Republic [36]. However, for the conservation of orchids, it is necessary to monitor not only meadows, but also forests in which several species of orchids occur [37]. A large part of the population of orchids in the Bohemian-Moravian Highlands is in protected areas and receive appropriate care. However, small localities with few plants, which are not of great interest to nature conservationists, are at significant risk [38].

This paper has focused on orchids, and it would be interesting to know whether the results can be generalized to other taxa. If so, the results would be very important for species conservation generally. The answer, however, is not that simple, as each case is species dependent. Thus, although the current approach would appear to have great potential, further research is needed.

## 4. Materials and Methods

The Czech Republic is in Central Europe and its flora are very well studied. The average altitude of the country is 450 m a.s.l. It is covered mainly by highlands of moderate altitude, whereas higher mountains occur at the borders with other countries (mainly in the north and south). The climate of the Czech Republic is typically temperate with cold, cloudy winters and hot summers. However, there are some regional and local differences due to the complex topography in this area [39]. Because the Czech Republic is a relatively small country in terms of latitudinal range, temperature and precipitation are mostly affected by local heterogeneity and altitude [40].

The Orchidaceae is used here as the model group, because of great species richness with about 20,000–35,000 species [41,42,43], many of which are threatened by extinction, great diversity of reproductive strategies [44] and extremely restricted distributions of small populations [23]. The five most abundant species in the Czech Republic (*Cephalanthera damasonium*, *Dactylorhiza fuchsii*, *Dactylorhiza majalis*, *Neottia ovata* and *Platanthera bifolia*) were chosen as a representative sample of orchid populations. The only species in this list that grows in shady places is *C. damasonium*, which is also the only one restricted to calcareous substrates. *P. bifolia*, *D. fuchsii* and *D. majalis* prefer open habitats or semi-shaded places. *D. fuchsii* and *D. majalis* prefer wetland habitats, whereas the rest occur in dry meadows or mesophilic grasslands. The only species occurring at high altitudes are *D. fuchsii* and *N. ovata*, the latter of which has a wide ecological amplitude in terms of habitat and altitude [45]. According to a Red List of the Czech Republic, *D. majalis* and *P. bifolia* are listed as endangered, category C3, while the others are listed as less threatened, category C4 [46].

Data from the NDOP database of the Nature Conservation Agency of the Czech Republic (https://portal.nature.cz/nd/, accessed on 18 February 2024), which includes the distributions of all the orchids in the Czech Republic, were used. This database includes a lot of information on the number of individuals in a population (e.g., individuals, flowering individuals, fertile individuals, sterile individuals, and so on) from many different contributors that may provide information about a population in various forms. Only those records that included valid species names and exact GPS coordinates were used. To avoid bias in the dataset and subsequent results, only those records that included the number of flowering and fertile individuals in a population were used because this gives clear information about the flowering part of the population. This is referred to as “flowering individuals”, as fertile plants will have flowered a couple of days previously. Records with the same locality ID number were pooled and all the duplicate records were deleted. After this, the dataset included a total of 2365 orchid populations. Using the “FREQUENCY” matrix function in MS Excel, the sites were categorized according to the number of flowering individuals in the population into groups with 1–10, 11–30, 31–100, 101–300, 301–1000, 1001–3000, and 3001–10,000 flowering individuals. Based on this dataset, bar graphs of the relationships between the number of populations and the number of flowering individuals in the population (the distribution of population sizes) were plotted for each of the species studied. All figures were produced in Microsoft Excel using some of its extensions.

We wanted to know whether it is better for the survival of a metapopulation to concentrate on conserving a few large populations, each of which will produce many propagules, or to concentrate on conserving many small populations, each of which will produce only few propagules.

Let us denote by *n* the log of the number of fertile individuals in a population (its “size”), and by *N* = *N*(*n*), the number of populations with *n* fertile individuals. Our empirical results indicate that *N* is a negative exponential of *n*:

(1) $$N\left(n\right)=ae^{-bn},$$

where *a* and *b* are parameters, *a* is proportional to the area of the region considered (e.g., the Czech Republic in this case) and *b* denotes the steepness of the negative exponential, with a large *b* denoting a steep decline, which means many small populations and few large ones, and vice versa.

If *k* is the average number of propagules produced by one individual in any of the populations considered, then the best estimate of the number of propagules produced by one population of size *n*, *P*_1,n_, is directly proportional to the number of fertile individuals in the population:

*P*_1,n_ = *nk*. (2)

Therefore, from (1) and (2), it follows that the number of propagules produced by all *N* populations, each containing *n* fertile individuals, is

(3) $$P_{N,n}=P_{1,n}.N=nkae^{-bn}.$$

For the ease of understanding the biological meaning of parameter *b*, randomly chosen figures for large and small *b* are depicted in Figure 2: large *b* means a steep decline in the corresponding negative exponential (Figure 2a), and yields a hump-shaped dependence of the total number of propagules produced by all populations of size *n*, *P*_N,n_, on the number of fertile individuals in the population (Figure 2b).

Small *b* indicates a slow decline in the negative exponential (Figure 2c). A decline in parameter *b* (from *b* = 0.001 to *b* = 0.0001) indicates a change from a hump-shaped dependence to a monotonously increasing dependence of the total number of propagules produced by all populations of size *n*, *P*_N,n_, on the number of fertile individuals in the population (Figure 2d).

## Data Availability

Restrictions apply to the availability of these data. Data were obtained from the Nature Conservation Agency of the Czech Republic and are available at https://portal.nature.cz/publik_syst/ctihtmlpage.php?what=3&nabidka=hlavni&variantaPrihlaseni=ISOP (accessed on 18 February 2024) with the permission of the Nature Conservation Agency of the Czech Republic.

## References

[1] Román-Palacios C., Wiens J.J. (2020). Recent responses to climate change reveal the drivers of species extinction and survival. Proc. Natl. Acad. Sci. USA.

[2] Humphreys A.M., Govaerts R., Ficinski S.Z., Lughadha E.N., Vorontsova M.S. (2019). Global dataset shows geography and life form predict modern plant extinction and rediscovery. Nat. Ecol. Evol..

[3] Jaureguiberry P., Titeux N., Wiemers M., Bowler D.E., Coscieme L., Golden A.S., Guerra C.A., Jacob U., Takahashi Y., Settele J. (2022). The direct drivers of recent global anthropogenic biodiversity loss. Sci. Adv..

[4] Balvanera P., Pfaff A., Viña A., García-Frapolli E., Merino L., Minang P.A., Nagabhatla N., Hussain S.A., Sidorovich A.A., Brondízio E.S., Settele J., Díaz S., Ngo H.T. (2019). Chapter 2.1. Status and Trends–Drivers of Change. Global Assessment Report of the Intergovernmental Science-Policy Platform on Biodiversity and Ecosystem Services.

[5] Newbold T., Hudson L.N., Hill S.L.L., Contu S., Lysenko I., Senior R.A., Börger L., Bennett D.J., Choimes A., Collen B. (2015). Global effects of land use on local terrestrial biodiversity. Nature.

[6] Štípková Z., Kindlmann P. (2021). Orchid extinction over the last 150 years in the Czech Republic. Diversity.

[7] Wright J., Pickering C. (2019). A continental scale analysis of threats to orchids. Biol. Conserv..

[8] Giam X., Bradshaw G.J.A., Tan H.T.W., Sodhi N.J. (2010). Future habitat loss and the conservation of plant biodiversity. Biol. Conserv..

[9] Le Roux J.J., Hui C., Castillo M.L., Iriondo J.M., Keet J.H., Khapugin A.A., Médail F., Rejmánek M., Theron G., Hirsch H. (2019). Recent anthropogenic plant extinctions differ in biodiversity hotspots and coldspots. Curr. Biol..

[10] Rejmánek M., Krahulec F., Grulich V. (2021). Jak rychle a proč vymírají rostliny v antropocénu. Živa.

[11] Moreira H., Kuipers K.J.J., Posthuma L., Zijp M.C., Hauck M., Huijbregts M.A.J., Schipper A.M. (2023). Threats of land use to the global diversity of vascular plants. Divers. Distrib..

[12] Sala O.E., Chapin F.S., Armesto J.J., Below E., Blomfield J., Dirzo R., Huber-Sanweld E., Huenneke L.F., Jackson R.B., Kinzig A. (2000). Global biodiversity scenarios for the year 2100. Science.

[13] Urban M.C. (2015). Accelerating extinction risk from climate change. Science.

[14] Wiens J.J. (2016). Climate-related local extinctions are already widespread among plant and animal species. PLOS Biol..

[15] Warren R., Price J., Graham E., Forstenhaeusler N., Vanderwal J. (2018). The projected effect on insects; vertebrates; and plants of limiting global warming to 1.5 °C rather than 2 °C. Science.

[16] Pigot A.L., Merow C., Wilson A., Trisos C.H. (2023). Abrupt expansion of climate change risk for species globally. Nat. Ecol. Evol..

[17] Mancini G., Santini L., Gazalis V., Akcakaya H.R., Lucas P.M., Brooks T.M., Foden W., Di Marco M. (2024). A standard approach for including climate change responses in IUCN Red List assessments. Conserv. Biol..

[18] Wiens J.J., Zelinka J. (2024). How many species will Earth lose to climate change?. Glob. Change Biol..

[19] Hanski I.A., Gilpin M.E. (1997). Metapopulation Biology: Ecology, Genetics, and Evolution.

[20] Hanski I. (1999). Metapopulation ecology. Oxford Series in Ecology and Evolution.

[21] Jersáková J., Malinová T. (2007). Spatial aspects of seed dispersal and seedling recruitment in orchids. New Phytol..

[22] Jacquemyn H., Brys R., Vandepitte K., Honnay O., Roldán-Ruiz I., Wiegand T. (2007). A spatially explicit analysis of seedling recruitment in the terrestrial orchid *Orchis purpurea*. New Phytol..

[23] Švecová M., Štípková Z., Traxmandlová I., Kindlmann P. (2023). Difficulties in determining distribution of population sizes within different orchid metapopulations. Eur. J. Environ. Sci..

[24] Bullock J.M., Clarke R.T. (2000). Long distance seed dispersal by wind: Measuring and modelling the tail of the curve. Oecologia.

[25] Paradis E., Baillie R.B., Sutherland W.J. (2002). Modeling large-scale dispersal distances. Ecol. Model.

[26] Nuttle T., Haefner J.W. (2005). Seed Dispersal in Heterogeneous Environments: Bridging the Gap between Mechanistic Dispersal and Forest Dynamics Models. Am. Nat..

[27] Jones F.A., Muller-Landau H.C. (2008). Masuring long-distance seed dispersal in complex natural environments: An evaluation and integration of classical and genetic methods. J. Ecol..

[28] Kotilínek M., Těšitelová T., Košnar J., Fibich P., Hemrová L., Koutecký P., Münzbergová Z., Jersáková J. (2020). Seed dispersal and realized gene flow of two forest orchids in a fragmented landscape. Plant Biol..

[29] Jersáková J., Kindlmann P. (2004). Zásady Péče o Orchidejová Stanoviště.

[30] Husband B.C., Barrett S.C.H. (1996). A metapopulation perspective in plant population biology. J. Ecol..

[31] Gaskett A.C., Gallagher R.V. (2018). Orchid diversity: Spatial and climatic patterns from herbarium records. Ecol. Evol..

[32] Charitonidou M., Halley J.M. (2020). What goes up must come down—Why high fecundity orchids challenge conservation beliefs. Biol. Conserv..

[33] Newman B.J., Ladd P., Batty A., Dixon K. (2007). Ecology of orchids in urban bushland reserves—Can orchids be used as indicators of vegetation condition?. Lankesteriana.

[34] Vogt-Schilb H., Geniez P., Pradel R., Richard F., Schatz B. (2013). Inter-annual variability in flowering of orchids: Lessons learned from 8 years of monitoring in a Mediterranean region of France. Eur. J. Environ. Sci..

[35] Wotavová K., Balounová Z., Kindlmann P. (2004). Factors affecting persistence of terrestrial orchids in wet meadows and implications for their conservation in a changing agricultural landscape. Biol. Conserv..

[36] Mokřady z.s. https://mokrady.wbs.cz/Mokrady---zakladni-informace.html.

[37] Štípková Z., Tsiftsis S., Kindlmann P. (2021). Distribution of orchids with different rooting systems in the Czech Republic. Plants.

[38] Čech L., Ekrt L., Ekrtová E., Jelínková J., Juřička J. *Dactylorhiza fuchsii* (Druce) Soó–Prstnatec Fuchsův v Kraji Vysočina. *Pobočka ČSO na Vysočině* 2017. www.prirodavysociny.cz.

[39] Palacký University Olomouc. “Climatic Conditions of the Czech Republic”. https://geography.upol.cz/soubory/lide/smolova/GCZ/GCZ_Klima.pdf.

[40] Štípková Z., Tsiftsis S., Kindlmann P. (2020). Pollination Mechanisms are Driving Orchid Distribution in Space. Sci. Rep..

[41] Dressler R.L. (1993). Phylogeny and Classification of the Orchid Family.

[42] Chase M.W., Cameron K.M., Barrett R.L., Freudebstein J.V., Dixon K.W., Kell S.P., Barrett R.L., Cribb P.J. (2003). DNA data and Orchidaceae systematics: A new phylogenetic classification. Orchid Conservation.

[43] Cribb P.J., Kell S.P., Dixon K.W., Barrett R.L., Dixon K.W., Kell S.P., Barrett R.L., Cribb P.J. (2003). Orchid conservation: A global perspective. Orchid Conservation.

[44] Steffelová M., Traxmandlová I., Štípková Z., Kindlmann P. (2023). Pollination strategies of deceptive orchids—A review. Eur. J. Environ. Sci..

[45] Průša D. (2005). Orchideje České Republiky.

[46] Grulich V., Chobot K. (2017). Red List of Threatened species of the Czech Republic. Vascular Plants. Příroda.

